# Survey of medium- and large-sized mammals in Atlantic Forest remnants of Conceição dos Ouros, Minas Gerais, Brazil

**DOI:** 10.3897/BDJ.10.e82139

**Published:** 2022-04-11

**Authors:** Ademir Henrique Vilas Boas, Iuri Veríssimo, Roberto Leonan Morim Novaes, Gabriel Cupolillo, Cecilia Siliansky de Andreazzi, Sócrates Fraga Costa-Neto, Ricardo Moratelli

**Affiliations:** 1 Fiocruz Mata Atlântica, Fundação Oswaldo Cruz, Rio de Janeiro, Brazil Fiocruz Mata Atlântica, Fundação Oswaldo Cruz Rio de Janeiro Brazil; 2 Programa de Pós-graduação em Biodiversidade e saúde, Instituto Oswaldo Cruz, Fundação Oswaldo Cruz, Rio de Janeiro, Brazil Programa de Pós-graduação em Biodiversidade e saúde, Instituto Oswaldo Cruz, Fundação Oswaldo Cruz Rio de Janeiro Brazil; 3 Programa de Pós-Graduação em Ecologia, Instituto de Biologia, Universidade Federal do Rio de Janeiro, Rio de Janeiro, Brazil Programa de Pós-Graduação em Ecologia, Instituto de Biologia, Universidade Federal do Rio de Janeiro Rio de Janeiro Brazil; 4 Laboratório de Biologia e Parasitologia de Mamíferos Silvestres Reservatórios, Instituto Oswaldo Cruz, Fundação Oswaldo Cruz, Rio de Janeiro, Brazil Laboratório de Biologia e Parasitologia de Mamíferos Silvestres Reservatórios, Instituto Oswaldo Cruz, Fundação Oswaldo Cruz Rio de Janeiro Brazil; 5 Centro de Ecología Funcional, Universidade de Coimbra, Coimbra, Portugal Centro de Ecología Funcional, Universidade de Coimbra Coimbra Portugal

**Keywords:** Camera traps, conservation, diversity, endangered species, Mammalia

## Abstract

Conceição dos Ouros is located in the Mantiqueira mountain range (elevation: 831‒1443 m a.s.l.), State of Minas Gerais, southeast Brazil. The largest two Atlantic Forest remnants of the Municipality of Conceição dos Ouros total more than 2,000 ha and the main vegetation type is seasonal semi-deciduous forest, isolated by a matrix of agricultural fields and pasture. The Municipality does not have any protected areas and is located in a highly fragmented region, albeit considered of special importance for the conservation of terrestrial vertebrates in the State of Minas Gerais. Due to a cooperation with the Municipality of Conceição dos Ouros to carry out a survey of the local biodiversity, in this study we present the results of the survey of medium- and large-sized terrestrial mammals from the two forest remnants in the region. Sampling was performed from July 2019 to August 2021 and comprised a camera trap survey, active searching including direct (e.g. carcass sightings) and indirect (e.g. footprints and faeces) evidence of species presence and interviews with residents. Twenty-nine native and two non-native species were documented. Ten species are in some category of threat of extinction at regional, national or global levels. This is the first survey of the terrestrial mammal fauna in the area of Conceição dos Ouros and results could be helpful in designing conservation strategies at the local scale.

## Introduction

Brazil has one of the highest numbers of mammalian diversity in the world, with more than 760 species documented (Abreu et al. 2021). The Atlantic Forest, a biodiversity hotspot and global priority area for conservation, contains more than 320 species of mammals, 90 of which are endemic to the biome (Paglia et al. 2012, Graipel et al. 2017, Quintela et al. 2020). The State of Minas Gerais, southeast Brazil, has 243 species of mammals, of which 70% are found in the Atlantic Forest and one third are unique to the biome (Ministério do Meio Ambiente et al. 2010). At least 44 mammal species that occur in Minas Gerais are threatened with extinction (Bastos-Neto et al. 2009, Copam - Conselho Estadual de Política Ambiental 2010), chief amongst them being primates and carnivores (Drummond et al. 2005, Bastos-Neto et al. 2009, Copam - Conselho Estadual de Política Ambiental 2010).

Habitat loss and fragmentation are the leading threats to mammals (Marinho-Filho and Machado 2006, Metzger 2009) and together with hunting, they mainly affect medium- to large-sized species (Graipel et al. 2017). These species are particularly affected because of their low reproductive rates and need for large home ranges and high resource availability (Trolle et al. 2007, Bocchiglieri et al. 2010). Thus, studies investigating the species richness, diversity, activity patterns and habitat use of mid- to large-sized mammals are essential to provide information for conservation strategies (Trolle et al. 2007, Oliveira et al. 2009, Bocchiglieri et al. 2010).

Biodiversity conservation strategies at a local scale should be designed, based on an understanding of the occurrence and distribution of species (Tobler et al. 2008) and the impacts of human activities on vulnerable species (Tobler et al. 2008, Cheyne et al. 2016, Rosas-Ribeiro et al. 2017). Thus, in this study, we present the results of a survey of medium- and large-sized terrestrial mammals from two Atlantic Forest remnants of the Municipality of Conceição dos Ouros in the State of Minas Gerais, southeast Brazil. The Conceição dos Ouros Municipality does not have any protected areas and is located in a highly fragmented region, albeit considered of special importance for the conservation of terrestrial vertebrates in the State of Minas Gerais (Drummond et al. 2005).

## Data resources

Individualised records of medium- and large-sized mammals from Conceição dos Ouros, MG, registered by camera trap, is available in Suppl. material 1.

## Material and Methods

### Study area

**Figure 1.**
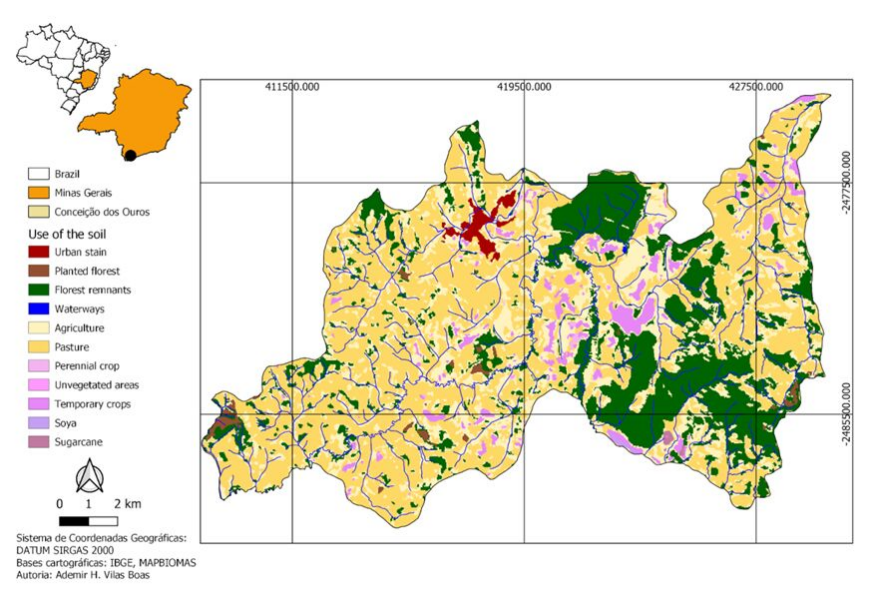
Forest remnants in the Municipality of Conceição dos Ouros, MG, with the characterisation of land use.

The study was conducted in the Municipality of Conceição dos Ouros, embedded in the Mantiqueira mountain range, southern Minas Gerais, at an elevation of 831‒1443 m a.s.l. Conceição dos Ouros has 2,062 ha of Atlantic Forest remnants, which comprise 11% of the municipality’s area (Fig. 1; Fundação SOS Mata Atlântica and Instituto Nacional de Pesquisas Espaciais 2014). The two fragments in the municipality > 500 ha were selected for the survey. These fragments were identified via satellite imagery and ground-truthing and are located on the Monte Alegre Farming Inc. farm, which contains most forest remnants of the Municipality.

**Figure 2.**
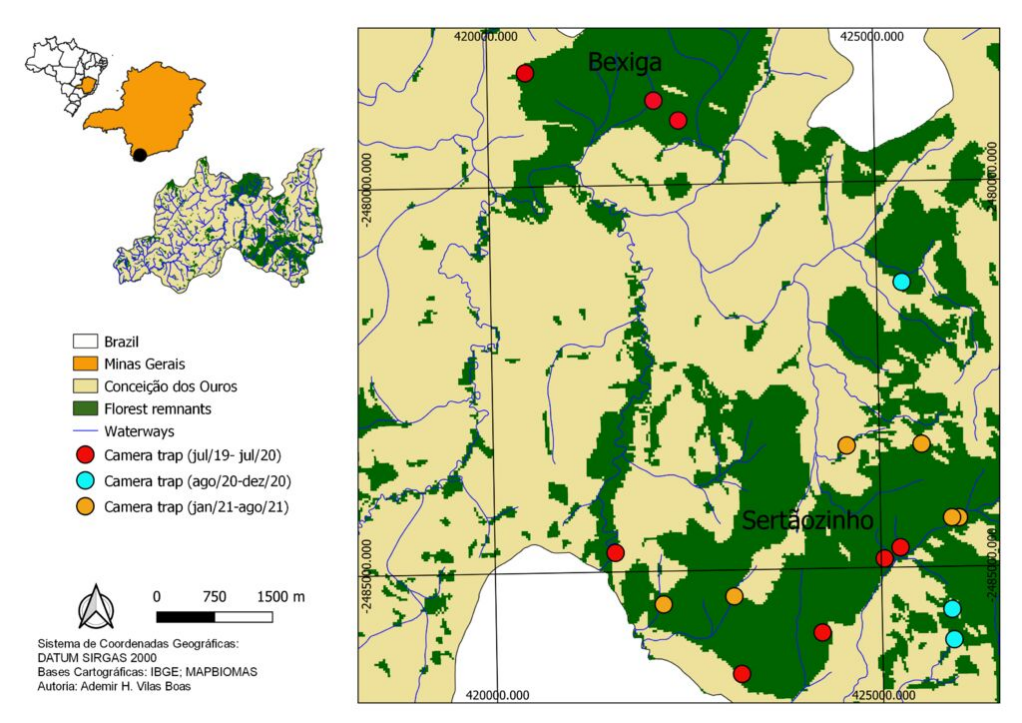
Distribution of camera traps for surveying mid- and large-sized mammals, by period (see caption), in the largest remnants of vegetation in the Municipality of Conceição dos Ouros, MG.

The Serra do Sertãozinho (22°28'4.68"S, 45°44'7.79"W; ca. 1,520 ha) and Mata da Bexiga (22°24'28.65"S, 45°45'27.51"W; ca. 576 ha) fragments were selected for mammal sampling (Fig. 2). Their predominant vegetation is seasonal semi-deciduous forest and there are several bodies of water in their interior emptying into the Sapucaí-Mirim River, which crosses the farm. In addition, the farm has a permanent preservation area (APP) under regeneration that potentially functions as a natural corridor between fragments. The fragments are surrounded by a matrix of modified habitat consisting of cassava (589 ha), corn (485 ha), coffee (398 ha) and sugar cane (10 ha) fields, pastures for livestock with a herd of ca. 11,490 head of cattle and a few areas at an early stage of natural regeneration (Faria 2017; Fig. 2).

The region’s climate is subtropical highland (Cwb; Köppen 1948) featuring well-defined rainy (October to March) and dry (April to September) seasons. The average annual precipitation is 1,500 mm and the average annual temperature is 18°C with temperature extremes nearing 0°C in winter and ~ 32°C in summer.

### Sampling

The study was conducted from July 2019 to August 2021 and comprised a camera trap survey, interviews with farm workers and residents of the fragments’ surroundings (Voss and Emmons 1996) and active searching including direct (e.g. visual and vocalisation records) and secondary (e.g. footprints and faeces) evidence of species presence (Carvalho Jr and Luz 2008, Becker and Dalponte 2013), in addition to occasional records, such as carcass sightings.

For the camera trap survey, eight Trophy Cam trail cameras (Bushnell, Overland Parks, KS, USA) were placed at ca. 40 cm above the ground. Cameras were installed randomly in spots where animals are expected to pass, such as trails, forest clearings and near small water bodies (Fig. 2), always baited with banana paste, oats, peanut butter and bacon. Camera traps remained operational throughout the study period and were repositioned every 90 days, totalling 765 sampling days for a sampling effort of 6,120 camera-days (Fig. 2). The images of all individuals of the same species detected by the same camera trap within a 1-h interval were treated as a single record. Active searches were conducted along trails in the interior of the fragments, totalling 263.33 km of trails covered over 90 sampling days randomly distributed within the study period. These suveys were conducted in the early morning (06:00-12:00 h) and late afternoon/early evening (16:00-00:00 h). Twenty-four interviews were conducted with farm workers and residents living near the fragments.These interviews aimed to survey the species that occur in the region and the human-animal interfaces (Suppl. materials 2, 3).

### Data analysis

All mid- to large-sized mammals with body weight > 1.0 kg were included (Chiarello 2000). We also included records of smaller animals in the region like *Didelphisalbiventris* Lund, 1840, *Didelphisaurita* (Wied-Neuwied, 1826), *Sylvilagus* sp. and *Coendouspinosus* (Cuvier, 1823) that could be reliably identified in the sampled area. Although it was not possible to distinguish *D.albiventris* and *D.aurita* based on camera trap records, the occurrence of these two species were confirmed by trap captures during the small mammal survey. Footprints were identified from Becker and Dalponte (2013) and Carvalho Jr and Luz (2008). When species detected by camera traps could not be reliably identified, images were submitted to experts in each taxonomic group for identification (see Acknowledgements). The nomenclature used for xenarthrans and marsupials followed different authors in Gardner (2008). For the others, the nomenclature follows Wilson and Reeder (2005).The classification into feeding habits was based on Paglia et al. (2012) and Magioli (2016). The conservation status of each species on a global, national and regional level was derived respectively from the IUCN (2021), the Red Book of Threatened Brazilian Fauna (Instituto Chico Mendes de Conservação da Biodiversidade 2018) and the Minas Gerais Red List (Copam - Conselho Estadual de Política Ambiental 2010).

The sampling effort was calculated by multiplying the number of camera traps by the number of active sampling days (unit: camera-days; Srbek-Araujo and Chiarello 2007). The species accumulation curve for mammal species of the two forest fragments combined in Conceição dos Ouros was constructed using EstimateS v. 9.1 (Colwell 2011). In addition, the capture success and photographic rate were also computed for each species. The estimated mammal species richness for Conceição dos Ouros was calculated using the Jackknife1 non-parametric richness estimator (Zahl 1977). Species diversity was calculated using the Shannon–Wiener (H’) Diversity Index and Simpson’s Heterogeneity Index. These analyses were all performed using PAST 3.0 software (Hammer et al. 2001).

## Results

The camera trap survey, interviews and other direct and secondary evidence revealed the occurrence of 31 species of wild terrestrial mammals, including 29 native and two non-native species distributed in six orders, 16 families and 26 genera (Table 1). The orders with the highest species richness were Carnivora (11 spp.) and Rodentia (6 spp.). The non-native species *Lepuseuropaeus* (European hare) and *Susscrofa* (wild boar) were included on the species list because they form populations living wild in the area. In addition, domestic dogs (*Canislupusfamiliaris*) and cattle (*Bostaurus*) were also recorded, but were not included on the species list because they are not wild animals. The species *Speothosvenaticus* (bush dog) and *Tamanduatetradactyla* (southern tamandua) were mentioned once each by residents during interviews, but due to a lack of additional evidence of their occurrence in the forest remnants of Conceição dos Ouros, we decided not to include them in the final species list. Four feeding habitats were identified (Table 1) and most species were omnivores (13 spp.), followed by herbivores (7 spp.), carnivores (6 spp.) and frugivores (5 spp.).

Twenty species were detected by camera traps, five by active searching and eight species were identified from interviews. Of the 20 species detected by the camera traps, *Didelphis* spp., *Dicotylestajacu* (collared peccary) and *Eirabarbara* (tayra) were the most frequent, accounting for 13.9%, 7.1% and 5.2% of all detections, respectively.

The Shannon-Wiener Diversity Index (H’) was 2.27 and 2.41 for the Mata da Bexiga and Serra do Sertãozinho fragments, respectively. The Simpson Index was 0.82 and 0.86 for Mata da Bexiga and Serra do Sertãozinho, respectively (Table 2). These results indicate that there are no differences in the structure of the mammal community between the two fragments and that the mammal fauna is diverse with no dominant species.

**Figure 3.**
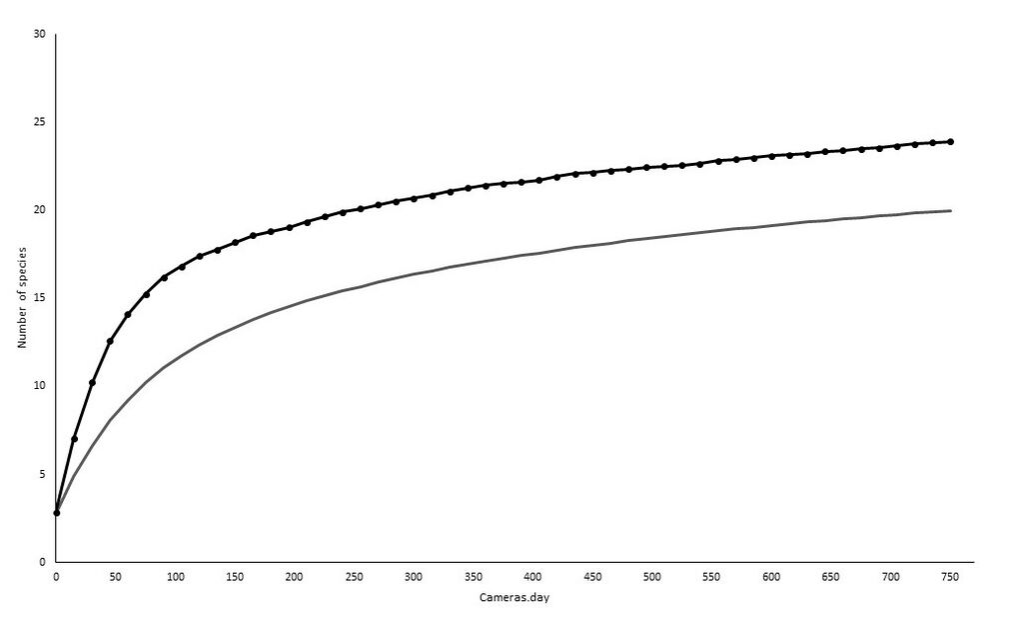
Accumulation curve of mid- and large-sized mammals recorded by camera traps in the remnants of vegetation in the Municipality Conceição dos Ouros, MG. The lower curve indicates the accumulation of observed species. The upper curve indicates the number of species estimated for the study area.

**Figure 4.**
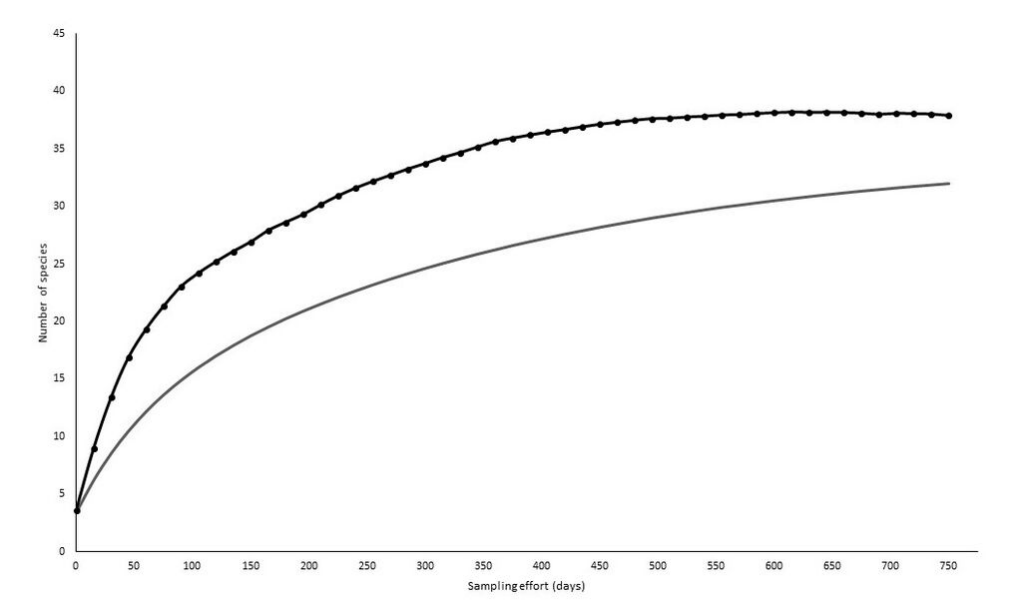
Accumulation curve of mid- and large-sized mammals recorded by camera traps, interviews and active searches in the remnants of vegetation in the Municipality Conceição dos Ouros, MG. The lower curve indicates the accumulation of observed species. The upper curve indicates the number of species estimated for the study area.

Total estimated species number of mid- and large-sized mammals was 22 with the Jackknife1 estimator using camera trap data only (Fig. 3). The species accumulation curve nearly reached saturation. In addition, when data were pooled from all sampling techniques (i.e. camera trapping, active searching and interviews), the estimated species richness for the Mata da Bexiga and Serra do Sertãozinho fragments was 40 with the Jackknife1 estimator (Fig. 4).

Of the 31 mammal species recorded, seven are coded as Vulnerable (*Alouattaguariba*, *Chrysocyonbrachyurus*, *Leopardusguttulus*, *Leoparduspardalis*, *Lontralongicaudis*, *Dicotylestajacu* and *Pumaconcolor*) and two as Endangered (*Callicebusnigrifrons* and *Callithrixaurita*) in the Minas Gerais Red List (Copam - Conselho Estadual de Política Ambiental 2010). In addition, six species are coded as Vulnerable (*Alouattaguariba*, *Callicebusnigrifrons*, *Chrysocyonbrachyurus*, *Leopardusguttulus*, *Pumaconcolor* and *Pumayagouaroundi*) and one species is listed as Endangered (*Callithrixaurita*) in the Red Book of Threatened Brazilian Fauna (Instituto Chico Mendes de Conservação da Biodiversidade 2018). At the global level, four species are currently listed as Vulnerable (*Alouattaguariba*, *Callicebusnigrifrons*, *Callithrixaurita* and *Leopardusguttulus*) and three as Near Threatened (*Chrysocyonbrachyurus*, *Lontralongicaudis* and *Sapajusnigritus*) in the IUCN (2021).

## Discussion

The richness of native mammals reported for Conceição dos Ouros comprises 3.8% of all mammal species recorded from Brazil, 11.9% of mammals documented for Minas Gerais and 11.1% of terrestrial mammals that occur in the Atlantic Forest (Paglia et al. 2012, Quintela et al. 2020). The observed species richness levels for Conceição dos Ouros (29 spp.) are similar to or higher than those reported in other surveys of small Atlantic Forest remnants of Minas Gerais— for example, Monte Belo (28 spp., Laurindo 2017), Pouso Alegre (22 spp., Costa et al. 2010), Viçosa (23 spp., Prado et al. 2008), Santa Rita do Sapucaí (15 spp., Eduardo and Passamani 2009) and Lavras (18 spp., Silva and Passamani 2009).

As observed by Ahumada et al. (2011), most of the recorded species are omnivores and herbivores. Most larger mammals tend to consume a greater variety of foods, combining high and low calorie foods (Cáceres et al. 2007). The presence of herbivores may reflect the availability of primary food resources in the studied areas, which support species with more specialist habits (Pinotti et al. 2015). The presence of carnivores, such as *P.concolor*, indicates a species-rich community. The record of several species of carnivorous, omnivorous and herbivorous mammals can be explained by the variety of landscapes and environments presented in the study area (Quadros and Cáceres 2001).

The Shannon-Wienner Diversity (H') and Simpson's Heterogeneity (1/D) Indices did not present significant differences, although the Simpson Index presents a significant value, demonstrating that there are no tendencies towards dominance of some species.The lower the anthropogenic interference, the higher the H', that is, the index of Diversity is related to the degree of disturbance to the environment. A study in small forest remnants, with areas ranging from 5.4 ha to 15 ha, carried out in the Atlantic Forest, in northern Paraná, recorded diversity indices ranging from 1.97 to 2.02, respectively. From these comparisons, it is observed that, in Atlantic Forest areas, values of the Shannon-Wienner Index greater than 2.0 are recorded only in heterogeneous habitats in a good state of conservation (Pires and Fabián 2013, Rossaneis 2014).

We highlight the occurrence of game species, such as the collared peccary (*Dicotylestajacu*), agouti (*Dasyproctaleporina*), lowland paca (*Cuniculuspaca*) and Brazilian guinea pig (*Caviaaperea*) that are extensively hunted in other localities. Hunting, even on a small scale, together with habitat fragmentation and the introduction of non-native species, is one of the major threats to mammal conservation (Primack and Rodrigues 2001, Costa et al. 2005). However, the high frequency of occurrence of these species indicates that illegal hunting activity in the region may be negligible. Moreover, it should be noted that our research team found no evidence of illegal hunting in the surveyed fragments during fieldwork and interviews. The location of the largest two vegetation fragments, within a private property, may be partly responsible for this scenario.

The presence of an area that serves as an natural corridor, structurally connecting the two fragments, probably contributes to the occurrence of mammal species that require large home ranges, such as the maned wolf and the puma (Costa et al. 2010). Even though the study area has a rich mammal fauna, species with greater requirements for habitat availability and quality like the South American tapir (*Tapirusterrestris*), the white-lipped peccary (*Tayassupecari*) and the northern muriqui (*Brachyteleshypoxanthus*) were not documented (Costa et al. 2010). In addition to hunting, the presence of non-native (*Lepuseuropaeus* and *Susscrofa*) and domestic (*Canislupusfamiliaris*) species in the forest remnants can have a major impact on wildlife fauna (Cuarón 2000, Campos et al. 2007). Besides competing for resources with the native forest rabbit (*Silvilagusbrasiliensis*), the European hare can also affect predation rates of native species (Buenavista and Palomares 2017). The occurrence of *Lepuseuropaeus* in southern Minas Gerais is apparently recent with the first documented record from 2008 in the Municipality of Pouso Alegre (Costa and Fernandes 2010). The presence of *Susscrofa* in the study area has been widely documented, indicating that the species has become established in the region of Conceição dos Ouros. Wild boars are amongst the world’s top 100 worst invasive species and one of the most widely distributed mammal species globally (Lowe et al. 2000). Their habit of wallowing and bathing daily in small water bodies causes damage to agriculture and the environment (Hegel and Marini 2013, Rosa et al. 2018). According to Rosa et al. (2018), eradication of wild boar in the Mantiqueira Range region is now economically impractical. Thus, continued control of the species is critical for the maintenance of habitats and conservation of the local mammal fauna. Moreover, non-native species, such as *Susscrofa*, act as reservoirs of infectious agents with zoonotic potential (Hayashi and Sanctis 2010) and can pose a risk to both human and environmental health. This threat can also originate from the presence of domestic dogs in fragments. Lessa et al. (2016) discussed the impact of domestic dogs on wildlife fauna resulting from transmission of disease to five species recorded in this study: *Chrysocyonbrachyurus*, *Cerdocyonthous*, *Leoparduspardalis*, *Pumaconcolor* and *Nasuanasua*. Finally, predation and competition pressure from domestic dogs on native fauna is another serious ecological consequence of their presence in natural habitats (Rangel and Neiva 2013, Doherty et al. 2017).

The occurrence of endangered species of large carnivores and primates in the study area highlights the need for developing conservation programmes for these species (Machado et al. 2008, Mendes et al. 2015). Moreover, several of the documented species are in some category of threat of extinction, further stressing the importance of the Atlantic Forest remnants of Conceição dos Ouros for the conservation of the regional mammal fauna (Konecny 1989, Yanosky and Mercolli 1989). It is also worth mentioning the large size of the largest two fragments, which together total more than 2,000 ha as opposed to over 83% of Atlantic Forest fragments which are < 50 ha (Ribeiro et al. 2009). Nevertheless, if the documented species are to maintain viable populations and play their roles in the ecosystem, it is crucial to increase wildlife surveillance and protection efforts and develop conservation and environmental education programmes locally. The results of this study can serve as the basis for designing conservation strategies for the local mammal fauna.

## Supplementary Material

AAF82955-0A65-5E75-BF41-39294071429F 10.3897/BDJ.10.e82139.suppl1 Supplementary material 1 Individualised records of mammals from Conceicao dos Ouros Data type Supplementary table Brief description Individualised records of medium- and large-sized mammals from Conceicao dos Ouros, registered by camera trap. File: oo_660291.xlsx https://binary.pensoft.net/file/660291 Vilas Boas et al.

45B7CB6D-EF32-5C77-B001-230D772A2101 10.3897/BDJ.10.e82139.suppl2 Supplementary material 2 Questionnaire applied to Fauna Survey Data type Questionnaire File: oo_660851.docx https://binary.pensoft.net/file/660851 Vilas Boas et al.

DEC51F05-8E67-5A9B-9307-CCC8EA4D9488 10.3897/BDJ.10.e82139.suppl3 Supplementary material 3 Free and Informed Consent Term Data type Consent Term File: oo_660849.docx https://binary.pensoft.net/file/660849 Vilas Boas et al.

## Figures and Tables

**Table 1.** Species of medium and large mammals recorded by camera traps (ct), capture (cp), carcass (ca), faeces (fe), footprints (fp), interviews (i) and visualisation (v) in the Atlantic Forest remnants of Conceição dos Ouros, MG, including their classification into feeding habits and conservation status at regional (Copam - Conselho Estadual de Política Ambiental 2010), national (Instituto Chico Mendes de Conservação da Biodiversidade 2018) and global (IUCN 2021) scales (LC = Least Concern; NT = Near Threatened; VU = Vulnerable; EN = Endangered, CR = Critically Endangered). Species classified as "Vulnerable" or "Endangered" on regional, national or global scales are marked with an asterisk (*). Introduced exotic species are marked with two asterisks (**).

**Taxa**	**Common name**	**Feeding habit**	**Record type**	**Conservation Status**		
				**MG**	**Brazil**	**Global**
**Artiodactyla**						
**Cervidae**						
*Mazamagouazoubira*	Grey brocket	Herbivore/Frugivore	ct, fp; i	-	LC	LC
**Tayassuidae**						
Dicotylestajacu*	Collared peccary	Omnivore	ct; fp; i	VU	LC	LC
**Suidae**						
Susscrofa**	Wild boar	Frugivore/Herbivore	ct; fp; i	Invasive	Invasive	LC
**Cingulata**						
**Dasypodidae**						
*Dasypusnovemcinctus*	Nine-banded armadillo	Omnivore/ Insectivore	ct; i	-	LC	LC
*Euphractussexcinctus*	Six-banded armadillo	Omnivore	ct; i	-	LC	LC
**Carnivora**						
**Canidae**						
*Cerdocyonthous*	Crab-eating fox	Omnivore	ct	-	LC	LC
Chrysocyonbrachyurus*	Maned wolf	Omnivore	ct; i; fe	VU	VU	NT
**Felidae**						
Leopardusguttulus*	Southern tigrina	Carnivore	ct, i	VU	VU	VU
Leoparduspardalis*	Ocelot	Carnivore	ct; i; ca	VU	LC	LC
Pumaconcolor*	Puma	Carnivore	ct; fp; i	VU	VU	LC
Pumayagouaroundi*	Jaguarundi	Carnivore	ct; i		VU	LC
**Mustelidae**						
*Eirabarbara*	Tayra	Omnivore	ct; i	-	LC	LC
*Galictisvittata*	Greater grison	Carnivore	i	-	LC	LC
Lontralongicaudis*	Neotropical otter	Carnivore	i	VU	LC	NT
**Procyonidae**						
*Nasuanasua*	Coati	Omnivore	ct; i	-	LC	LC
*Procyoncancrivorus*	Crab-eating raccoon	Frugivore/ Omnivore	ct; i	-	LC	LC
**Didelphimorphia**						
**Didelphidae**						
*Didelphisalbiventris*	White-eared opossum	Omnivore	cp; i	-	LC	LC
*Didelphisaurita*	Black-eared opossum	Omnivore	cp; i	-	LC	LC
**Lagomorpha**						
**Leporidae**						
Lepuseuropaeus**	European hare	Herbivore	i	Invasive	Invasive	LC
*Sylvilagus* sp.	Tapeti	Herbivore	i	-	-	LC
**Pilosa**						
**Bradypodidae**						
*Bradypusvariegatus*	Brown-throated sloth	Herbivore	i	-	LC	LC
**Primates**						
**Atelidae**						
Alouattaguaribaclamitans*	Southern brown howler	Herbivore	i; v	VU	VU	LC
**Callitrichidae**						
Callithrixaurita*	Buffy-tufted marmoset	Omnivore	i	EN	EN	VU
*Callithrixpenicillata*	Black-tufted marmoset	Omnivore	e; v	-	LC	LC
**Cebidae**						
*Sapajusnigritus*	Black capuchin	Omnivore	ct; i; v	-	LC	NT
**Pitheciidae**						
Callicebusnigrifrons*	Black-fronted titi	Omnivore	i	EN	VU	VU
**Rodentia**						
**Caviidae**						
*Caviaaperea*	Brazilian guinea pig	Herbivore	i	-	LC	LC
*Hydrochoerushydrochaeris*	Capybara	Herbivore	ct; fp; i; fe; v	-	LC	LC
**Cuniculidae**						
*Cuniculuspaca*	Lowland paca	Frugivore/Herbivore	ct; i	-	LC	LC
**Dasyproctidae**						
*Dasyproctaleporina*	Red-rumped agouti	Frugivore/Herbivore	i	-	LC	LC
**Erethizontidae**						
*Coendouspinosus*	Paraguayan hairy dwarf porcupine	Frugivore	i	-	LC	LC

**Table 2.** Shannon–Wiener Diversity Index (H’) and Simpson Index for the Mata da Bexiga and Serra do Sertãozinho fragments.

	estimator	observed	estimated	standard error	CI (95% lower)	CI (95% upper)
Bexiga						
	Shannon	2.272	2.363	0.108	2.272	2.576
	Simpson	0.822	0.826	0.018	0.822	0.861
Sertãozinho						
	Shannon	2.41	2.482	0.088	2.41	2.654
	Simpson	0.861	0.864	0.011	0.861	0.885

## Hosting institution

Fundação Oswaldo Cruz - Fiocruz
