# Supplementary material for: Survey of medium- and large-sized mammals in Atlantic Forest remnants of Conceição dos Ouros, Minas Gerais, Brazil
Source: Biodivers Data J. 2022 Apr 11;10:e82139. doi: 10.3897/BDJ.10.e82139 (PMC9016035; doi:10.3897/BDJ.10.e82139)
Supplement: Supplementary material 2 — Questionnaire applied to Fauna Survey [file bdj-10-e82139-s002.docx]

Model of a questionnaire related to fauna, applied to the residents of Conceição dos Ouros

| Data: ____/ ____/ _______  Interviewer name: ______ |
| --- |

| **Interviewee's profile** |
| --- |
| Interviewee's name: (Optional) |
| Gender: ( ) Male ( ) Female |
| Age: |
| Profession: |
| ( ) Salaried Position:_____  ( ) Farmer ( ) Self-employed  ( ) Teacher ( ) Student  ( ) Another. Which one? ________ |
| Schooling: |
| ( ) Incomplete Elementary School ( ) Complete Elementary School  ( ) Incomplete High School ( ) Complete High School  ( ) Incomplete Higher Education( ) Complete Higher Education |
| Location where you live? |
| ( ) Conceição dos Ouros rural area( ) Zonal urban de Conceição dos Ouros  ( ) Other municipalities |

| **Issues** |
| --- |
| **1.** Have you ever seen any wild animals in the forests of the municipality? ( ) yes ( ) no Which? |
| _________________________________________________________________________________________________________________________________________________________________________________________________________________________________ |
| **2.** What animals existed 10 years ago and today there are no more here? |
| ___________________________________________________________________________________________________________________________________________________________________________________________________________________________ |
| **3.** Which animals from the region do the people here usually use to: |
| a) Food use: |
| ___________________________________________________________________________ |
| b) Use of leather, fur and skin: |
| _________________________________________________________________________ |
| (c) Medicinal use: |
| ___________________________________________________________________________ |
| d) Use for handicrafts: |
| _________________________________________________________________________ |
| e) Mystical activities: |
| ___________________________________________________________________________ |
| f) Embellishment: |
| _________________________________________________________________________ |
| g) Creation. What? |
| ___________________________________________________________________________ |
| h) Crossing. What? |
| ___________________________________________________________________________ |
| i) As a pet. What? |
| __________________________________________________________________________________________________________________________________________________ |
| **4.** How do you acquire, or have ever purchased, wild animals for consumption or other purpose? |
| () trade lodges () commercial stores () hunting myself () other street walkers  ( ) never |
| 5.Do you believe that wild animals within the forests of the municipality are well protected? ( ) Yes ( ) No |
| Because__________________________________ |
| **6.** Do you believe that there is or exist in the forests of the municipality some wild animal that causes problems the neighboring properties? |
| ( ) Yes ( ) No |
| Because_________________________________ |
| **6.1.** If so, how to solve the problem? |
| __________________________________________________________________________________________________________________________________________________ |
| **7.** Have you reported any environmental problems in the region? ( ) Yes ( ) No Which (ais)? |
| __________________________________________________________________________________________________________________________________________________  _________________________________________________________________________ |
