# Supplementary material for: Survey of medium- and large-sized mammals in Atlantic Forest remnants of Conceição dos Ouros, Minas Gerais, Brazil
Source: Biodivers Data J. 2022 Apr 11;10:e82139. doi: 10.3897/BDJ.10.e82139 (PMC9016035; doi:10.3897/BDJ.10.e82139)
Supplement: Supplementary material 3 — Free and Informed Consent Term [file bdj-10-e82139-s003.docx]

FREE AND INFORMED CONSENT TERM

You are being invited (a) to participate in the research entitled Diversity of mammals of the Atlantic Forest of Conceição dos Ouros-MG, under the responsibility of researchers Me. Ademir Henrique Vilas Boas and Dr. Ricardo Moratelli.

In this research we seek to understand the perception of community residents in relation to the environment. The Consent and Free Clarification Form will be obtained by researcher Me. Ademir Henrique Vilas Boas during the interview we will do with the resident. In your participation you should answer some questions about the environment through the interview in the form of a questionnaire.

At no time will you be identified. The search results will be published and your identity will still be preserved.

You will not have any financial expenses or gains for participating in the survey. The risks consist of the possibility of you feeling embarrassed (a) when answering our questions, but all care has been taken to ensure that this does not happen. The benefits will be to better integrate you residents with each other, even momentarily, through the activities, which can result in some gain for yourselves, in addition, will make you reflect, on the topics discussed and exposed and on the very words of people during the discussions. You are free to stop participating in the survey at any time without any prejudice or coerto.

An original way of this Consent and Free Clarification Form will stay with you.

Any questions about the survey, you can contact: Me. Ademir Henrique Vilas Boas by e-mail: [ademirvilasboas@hotmail.com](mailto:ademirvilasboas@hotmail.com) or phone (35) 9 9917-4489 and Oswaldo Cruz Foundation - Oswaldo Cruz Institute: Av. Brazil, 4365 - Manguinhos, Rio de Janeiro - RJ, ZIP Code: 21040-900. You can also contact fiocruz's Research Ethics Committee (CEP Fiocruz), e-mail: [etica@fiocruz.br](mailto:etica@fiocruz.br), phone (21) 3882-9000 (Extension 9011) or by fax (21) 2561-4815.

Rio de Janeiro, ________ from _________

____________________________________________________

Researchers' Signature

I agree to participate in the above project voluntarily after being properly clarified.

_______________________________________

Survey participant
